# Differential associations of visual memory with hippocampal subfields in subjective cognitive decline and amnestic mild cognitive impairment

**DOI:** 10.1186/s12877-022-02853-7

**Published:** 2022-02-24

**Authors:** Yanlu Huang, Lin Huang, Yifan Wang, Yuchen Liu, Chun-Yi Zac Lo, Qihao Guo

**Affiliations:** 1 Department of Gerontology, Shanghai Jiao Tong University Affiliated Sixth People’s Hospital, Shanghai, China; 2 Institute of Science and Technology for Brain Inspired Intelligence, Fudan University, Shanghai, China

**Keywords:** Subjective cognitive decline (SCD), Amnestic mild cognitive impairment (aMCI), Hippocampal subfield volumes, Visual memory

## Abstract

**Background:**

Although previous studies have demonstrated that the hippocampus plays a role in verbal memory, the role of hippocampal subfields in visual memory is uncertain, especially in those with preclinical Alzheimer's disease (AD). This study aimed to examine relationships between hippocampal subfield volumes and visual memory in SCD (subjective cognitive decline) and aMCI (amnestic mild cognitive impairment).

**Methods:**

The study sample included 47 SCD patients, 62 aMCI patients, and 51 normal controls (NCs) and was recruited from Shanghai Jiao Tong University Affiliated Sixth People's Hospital. Visual memory was measured by the subtests of BVMT-R (Brief Visuospatial Memory Test-Revised), PLT (Pictorial Learning Test), DMS (Delayed Matching to Sample), and PAL (Paired Associates Learning). Hippocampal subfield volumes were estimated using FreeSurfer software (version 6.0). We modeled the association between visual memory and relative hippocampal subfield volumes (dividing by estimated total intracranial volume) using Pearson's correlation and linear regression.

**Results:**

Compared with the NC group, patients with SCD did not find any relative hippocampal subregion atrophy, and the aMCI group found atrophy in CA1, molecular layer, subiculum, GC-ML-DG, CA4, and CA3. After adjusting for covariates (age, sex, and APOE ε4 status) and FDR (false discovery rate) correction of p (q values) < 0.05, in NC group, DMS delay matching scores were significant and negatively associated with presubiculum (r = -0.399, FDR q = 0.024); in SCD group, DMS delay matching scores were negatively associated with CA3 (r = -0.378, FDR q = 0.048); in the aMCI group, BVMT-R immediate recall scores were positively associated with CA1, molecular layer, subiculum, and GC-ML-DG (r = 0.360–0.374, FDR q < 0.036). Stepwise linear regression analysis confirmed the association.

**Conclusions:**

Our results indicate a different and specific correction of visual memory with relative hippocampal subfield volumes between SCD and aMCI. The correlations involved different and more subfields as cognitive decline. Whether these associations predict future disease progression needs dynamic longitudinal studies.

**Supplementary Information:**

The online version contains supplementary material available at 10.1186/s12877-022-02853-7.

## Background

Alzheimer’s disease (AD) is a disease with a long prodromal phase, and there is a continuous progression from the preclinical stage through the prodromal stage and finally to dementia [1]. Amnestic mild cognitive impairment (aMCI) is a mild impairment in episodic memory function and is associated with a higher likelihood of developing dementia. More recently, interest has grown in subjective cognitive decline (SCD), regarded as at risk of the stage before developing aMCI and AD dementia [2]. People with SCD experience a subjective decline in any cognitive domain, but findings from objective examinations are within the normal range on standardized neuropsychological tests [3]. SCD with memory loss as a prominent perception is more likely to be preclinical SCD in AD [4]. SCD and aMCI had heterogeneity in disease manifestation and progression [5]. AD-like changes in amyloid beta (Aβ), tau brain accumulation, and neuroimaging increase the risk of SCD and aMCI future progression to AD [6–13].

Nevertheless, it is challenging to find evidence of changes from objective indicators in preclinical AD. Structural alterations are considered to precede objective psychological indicators. The hippocampus was thought to be an essential and available biomarker of neurodegeneration in prodromal AD, as this region was strongly associated with short-term and long-term memory [14]. However, several cross-sectional studies have not found alterations in the hippocampal subfields volume of SCD [15, 16]. Longitudinal analyses demonstrated that SCD is associated with longitudinal hippocampal atrophy [17, 18]. Some studies have attempted to find an intrinsic link between hippocampal or hippocampal subfields and memory, suggesting that it may be related to changes or progression at the early stage of AD [16, 19].

The association between the hippocampus and episodic memory is well established. However, most studies have focused on verbal episodic memory while ignoring visual episodic memory. Visual recognition memory performance was always found better than auditory recognition memory performance [20]. However, one study found visual memory decreased for days, while auditory memory was more resistant to information loss over time substantially [21]. A longitudinal study indicated a decline in visual memory measured by paired associates learning (PAL) and delayed matching to sample (DMS) tests could successfully distinguish different types of MCI and predicted mental outcomes [22]. Moreover, another study thought Brief Visuospatial Memory Test-Revised (BVMT-R) delayed recall was sensitive to amyloid positivity in nondemented older adults [23]. Recent research indicated that continuous Aβ measures correlated more significantly with graphic memory scores than verbal memory scores in dementia or neurodegeneration subjects, but not in non-dementia or non-neurodegeneration groups [24].

Hippocampal subfields mainly include the cornu ammonis (CA)1, CA2–3, CA4, dentate gyrus (DG), presubiculum, and subiculum. Studies on SCD, aMCI, and AD all showed that the volume of the left subiculum was most strongly and actively correlated with Auditory Verbal Learning Test (AVLT) measures [16]. The right hippocampal volume has been found to be more strongly associated with visual memory, while the left hippocampal volume has been more strongly associated with verbal memory [25]. Another study reported that anterior hippocampus subregion volumes (head) correlated with verbal memory, while some anterior and many posterior hippocampus subregion volumes (body and tail) correlated with visual memory scores. Visual memory was thought associated with hippocampal volumes bilaterally, especially DG and Posterior CA [26]. Previous studies found subiculum and CA1 was strongly associated with Complex Figure Delayed Recall and Free and Cued Selective Reminding Test Delayed Recall abilities in older adults [27]. A comparison study found that the BVMT-R learning slope was more strongly positively associated with hippocampal volume than the Hopkins Verbal Learning Test-Revised (HVLT-R) learning slope. The authors thought non-verbal memory measures might have higher diagnostic value, particularly in individuals at elevated risk for Alzheimer’s disease [28]. Therefore, understanding the relationships between visual episodic memory measures and hippocampal volumes may help early detection and prevention of AD. Identifying sensitive tests to hippocampal volume changes may offer a promising approach to diagnosing presymptomatic AD stages. However, a more in-depth analysis of subfields differentiation, especially the association with visual memory, lacks data in SCD patients. Furthermore, whether this association is consistent with that of aMCI remains unknown.

To fill the gap, the purpose of the present study was to (1) investigate relationships between visual memory and hippocampal subfield volumes in patients with SCD and aMCI. We also (2) evaluated the differences in visual memory performance and hippocampal subfield volumes compared to normal controls (NCs). We expected to find specific relationships between visual memory and hippocampal subfield volumes in patient groups relative to healthy controls.

## Methods

### Participants

We recruited 160 right-handed Chinese participants, including 47 SCD patients, 62 aMCI patients, and 51 NC subjects, from January 2020 to February 2021. The participants with SCD and aMCI were recruited from the memory clinic of the Gerontology Department of Shanghai Jiao Tong University Affiliated Sixth People's Hospital. The NC group was recruited from the Shanghai community through advertisements.

Participants meeting the following criteria were included: (a) over 60 years old; (b) having at least six years of formal education; (c) having normal vision and hearing and being able to complete a series of neuropsychological measurements; (d) non-dementia, the score of MMSE (Chinese Version Of The Mini-Mental State Examination) [29] ≥ 24; (e) no history of stroke, psychiatric disorder, cancer, substance abuse, epilepsy, brain tumor, Parkinson's disease, encephalitis, traumatic brain injury, or diseases that affect brain function; and (f) no physical problems that are not appropriate for MRI scan.

The NC patients were required to meet the following research criteria: (a) no memory complaints; (b) their performance on the MMSE, MoCA-B (Chinese version of the Montreal Cognitive Assessment Basic) [30], and AVLT [31] were within normal ranges (adjusted for age and education); (c) a CDR (Clinical Dementia Rating) [32] score of 0; and (d) a score of 12 or less on the Hamilton Depression Rating Scale within the past two weeks [33].

The diagnostic criteria for patients with SCD were based on SCD plus features published in 2014 [4] and included (a) subjective decline in memory rather than other domains; (b) onset of SCD with the last five years; (c) age of onset at 60 years or older; (d) concerns (worries) associated with SCD; (e) feeling of worse performance than others of the same age group; (f) normal performance on neuropsychological scales (MMSE, MoCA-B, AVLT) after age, and education adjustments that did not reach the criteria for MCI or dementia.

The patients were diagnosed with aMCI using criteria proposed by Jak/Bondi [34], which included (a) cognitive concern or complaints during the last year; (b) MMSE score above the cutoff value (> 24/30); (c) objective memory impairment assessed by long-delay free recall and AVLT identification at least 1.0 standard deviation (SD) below normal for age and education; (d) minor impairment in maintaining activities of daily living or instrumental activities of daily living; (e) absence of dementia, according to the NIA-AA criteria.

### Neuropsychological measures

This study focuses specifically on visual memory: (1) Traditional face-to-face neuropsychological assessments: BVMT-R [35] and PLT (Pictorial Learning Test) designed by Qihao Guo [36]; (2) CANTAB (Cambridge Neuropsychological Test Automated Battery) [37]: DMS and PAL were administered with a touch screen pad under the user manual’s instructions. For more details, please search the website: http://www.cantab.com. These visual memory tests were not used in the diagnostic process.

#### BVMT-R

The BVMT-R is a nonverbal episodic memory measure. The test stimuli consisted of six geometric shapes presented over three learning trials. Each learning session was presented for 10 s. Five and twenty minutes after the three learning trials, the subjects were asked to draw spontaneously and identify as many shapes as possible. The analyzed measure was the immediate recall score (total scores across trials 1–3) (BVMT-R IR).

#### PLT

The PLT is a short cognitive performance test assessing memory deficits. The patient was shown a board depicting 12 pictures (bell, banana, key, strawberry, fish, flower, dog, bicycle, chair, umbrella, teacup, and scissors) that were to be named and memorized at the same time. Then, the subjects were asked to recall and recognize as many pictures as they could remember after 10 min. The analyzed measure was the delayed recall score (PLT-DR).

#### DMS

The DMS assesses both simultaneous and short-term visual memory. The participant was shown an abstract visual pattern (i.e., the sample) and four similar patterns. The four patterns appeared together with the sample after a 4-s or 12-s delay. The participant was instructed to select the pattern that exactly matched the sample. The analyzed measure was the DMS percent correct (all delays) (DMSPCAD).

#### PAL

The PAL test assesses visuospatial episodic memory and learning. Six white boxes were displayed on a computer screen and were “opened” in a randomized order. Depending on the particular stage, 1, 2, 3, 6, or 8 figures were displayed in the boxes. The participant must select the box in which the pattern was initially located. If the participant makes an error, the boxes are opened in sequence again to remind the participant of the locations of the patterns. The analyzed measure was the PAL first attempt memory score (PALFAMS).

For descriptive purposes, we also report scores from Addenbrooke’s Cognitive Examination III (ACE-III) [38], Animal Verbal Fluency Test (AFT) [39], Boston Naming Test (BNT) [40], Shape Trail Test (STT) [41], Symbol Digit Modalities Test (SDMT) [42]. Experienced neurologists assessed the cognitive functions of all the subjects.

### Structural MRI parameters

Imaging was performed using a 3.0-Tesla scanner (SIEMENS MAGNETOM Prisma 3.0 T, Siemens, Erlangen, Germany). T1-weighted MRI scans were obtained in the sagittal plane by using a magnetization-prepared rapid acquisition gradient echo sequence with the following parameters: TR = 3000 ms, TE = 2.56 ms, FA = 7°, inversion time (TI) = 1100 ms, matrix = 320 × 320, slice number = 208 slices, slice thickness = 0.8 mm and voxel size = 0.8 × 0.8 × 0.8 mm^3^.

The 3D T1 MRI images were processed using the FreeSurfer image analysis software package (version 6.0, http://freesurfer.net/) [43] for hippocampal segmentation and volume calculation. The main steps were as follows: (1) conversion of the original DICM format to mgz format by running the dcm2nii and mri_convert commands; (2) cephalometric correction to remove non-brain tissue; (3) alignment to the Talairach standard spatial system; (4) probabilistic brain mapping to segment subcortical and cortical structures; and (5) segmentation of the hippocampus into 12 subregions according to the FreeSurfer built-in module. We focused on the volume of the CA1, presubiculum, parasubiculum, molecular layer, granule cell molecular layer of the dentate gyrus (GC-ML-DG), CA3, CA4, and hippocampal amygdala transition area (HATA) and excluded the hippocampal tail, hippocampal fissure, and fimbria from the analysis [44]. We averaged the left and right hippocampal subfields to decrease the number of multiple comparisons performed. Moreover, the recon-all command was used to obtain an estimated total intracranial volume (eTIV). Finally, relative hippocampal subfield volumes were calculated by dividing hippocampal subfield volumes by eTIV (accounting for differences in subject head volume) and multiplying by 10,000.

**Fig. 1:**
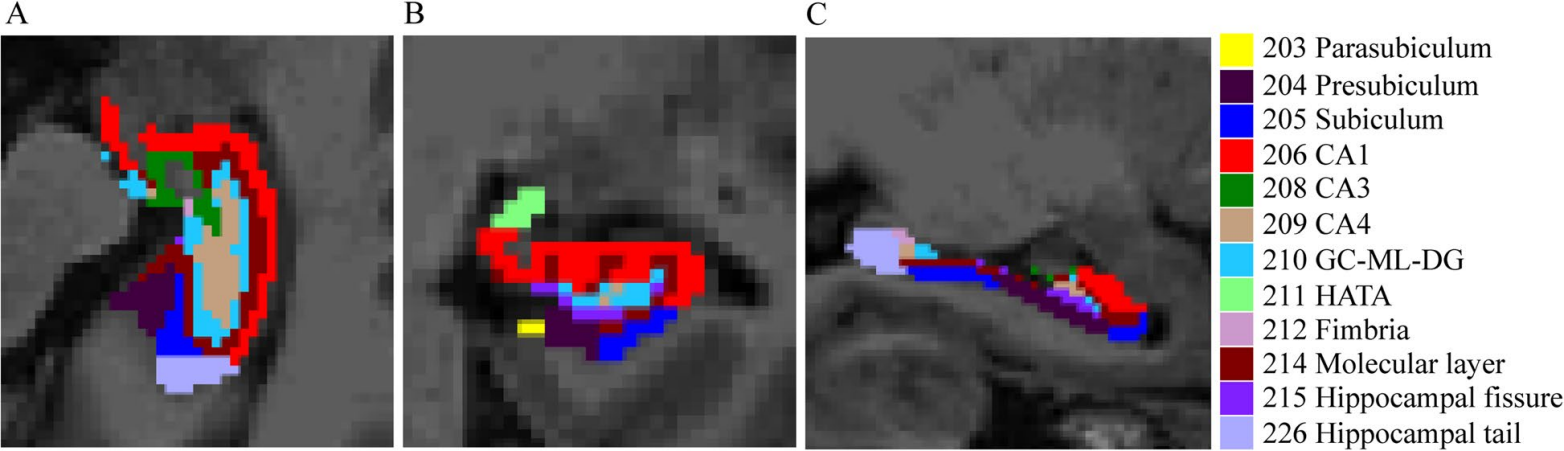
Visualization of hippocampal subfield segmentation. **A** Axial view, **B** Coronal view, **C** Sagittal view. CA, cornu ammonis; GC-ML-DG, granule cell molecular layer of dentate gyrus; HATA, hippocampal amygdala transition area

Figure 1 shows a visual depiction of hippocampal subfields.

### APOE genotyping

Two milliliters of peripheral venous blood were collected from all subjects, anticoagulated with EDTA, and stored in a freezer at -80 °C. After collecting blood samples from all subjects, genomic DNA was extracted with the Spin Columns DNA Isolation Kit (Generay Biotech Co., Ltd, Shanghai, CN) following the manufacturer’s protocol. In accordance with the manufacturer's instructions, two decisive genetic loci of APOE, rs7412 and rs429358, were detected by single nucleotide polymorphism detection reaction technology.

### Statistical analysis

Statistical analyses were performed using IBM SPSS Statistics for Windows (Version 23.0). Pearson’s chi-square tests were conducted for categorical variables. Group differences in demographic data were assessed using one-way independent ANOVA. Kruskal-Wallis tests were used if the homogeneity of variance was violated. We used principal component analysis (PCA) followed by varimax rotation to generate cognitive domain components from the neurocognitive test results. Pearson coefficients were further performed to test correlations between neuropsychological tests. A multivariate analysis of covariance (MANCOVA) was conducted to assess the effect of continuous and categorical variables (age, education, diagnosis, sex, and APOE ε4 status) on relative hippocampal subfield volumes. Then the univariate analysis of covariance (ANCOVA) was performed to assess the relationships between diagnosis and relative hippocampal subfield volumes with the significant variables as covariates. Bonferroni-corrected post hoc tests were conducted for between-group comparisons of continuous data. The significance level of all two-tailed p values was 0.05. Pearson correlation coefficients and stepwise linear regression analysis were performed to examine relationships between relative hippocampal subfield volumes and visual memory scores controlling for the above covariates. Heatmaps were created using Excel. Multiple comparison correction for correlation analysis was performed using the Benjamini-Hochberg procedure with a threshold false discovery rate (FDR) of 0.05.

## Results

### Demographics and neuropsychological test results

The detailed demographic and clinical characteristics of the participants are shown in Table 1. The study included 160 individuals (58.8% females), including 51 in the normal control group, 47 in the SCD group, and 62 in the aMCI group. Their mean age was 67.94 ± 4.87 years, and the average length of their education was 12.00 ± 2.66 years. No significant differences were observed for age, education, or sex distribution among the three groups. The percentage of APOE ε4 carriers was significantly different among the three groups and gradually increased across the NC (13.7%) to SCD (21.3%) to aMCI (40.3%) groups. In the neuropsychological tests, the aMCI group exhibited significant cognitive function deficits in general cognitive, language, attention/executive, and memory function compared to the NC group. Between the NC and SCD groups, the SCD group had significantly lower scores than the NC group on general cognitive function (MoCA-B and ACE-III), language function (AFT), attention/executive function (SDMT), and memory function (AVLT-DR, BVMT-R IR, PLT-DR, and PALFAMS).

**Table 1:** Demographics and neuropsychological tests for NC, SCD, aMCI

Index	NC *n* = 51	SCD *n* = 47	aMCI *n* = 62	Test statictics	p value
Age (y)	67.98 ± 5.01	66.81 ± 4.67	68.76 ± 4.81	2.173	0.117
Education (y)	12.65 ± 2.91	11.83 ± 2.19	11.61 ± 2.70	2.334	0.100
Sex (% Female)	27 (52.9)	34 (72.3)	33 (53.2)	5.073	0.079
APOE (% ε4)	7 (13.7)	10 (21.3) *	25 (40.3) **†	11.075	0.004
**General Cognition**					
MMSE (range 24–30)	28.61 ± 1.29	27.93 ± 1.41	26.13 ± 1.53**†	46.336	< 0.001
MoCA-B (range 18–30)	27.06 ± 1.33	25.83 ± 2.11*	22.77 ± 2.57**†	75.078	< 0.001
ACE-III (range 60–97)	87.57 ± 4.12	81.19 ± 5.38 **	73.50 ± 6.03**†	99.939	< 0.001
**Language Function**					
AFT (range 9–29)	19.26 ± 3.50	17.34 ± 4.29*	14.55 ± 3.62**†	22.046	< 0.001
BNT (range 17–30)	25.67 ± 2.61	25.06 ± 2.69	22.82 ± 3.21**†	15.492	< 0.001
**Attention/Executive Function**					
STT-A (range 21–140)	45.02 ± 14.46	51.02 ± 14.24	56.39 ± 19.13**	17.344	0.002
STT-B (range 21–238)	118.55 ± 28.03	117.6 ± 27.85	147.05 ± 38.35**†	20.993	< 0.001
SDMT (range 13–68)	42.28 ± 8.63	36.79 ± 9.03*	30.97 ± 10.37**†	20.103	< 0.001
**Veral Memory**					
AVLT-IR (range 5–29)	19.29 ± 4.16	17.38 ± 4.44	12.16 ± 2.89**†	70.266	< 0.001
AVLT-DR (range 0–12)	6.16 ± 2.19	5.11 ± 1.97*	1.73 ± 1.37**†	90.483	< 0.001
AVLT-Re (range 11–24)	22.18 ± 1.32	21.45 ± 2.24	17.81 ± 2.35**†	82.978	< 0.001
**Visual Memory**					
BVMT-R IR (range 2–36)	21.92 ± 7.08	18.34 ± 7.00*	15.24 ± 6.17**	13.842	< 0.001
PLT-DR (range 0–12)	7.86 ± 1.84	6.81 ± 2.14*	5.61 ± 2.31**†	15.868	< 0.001
DMSPCAD (range 27–100)	79.10 ± 12.81	77.17 ± 16.85	69.65 ± 16.68**†	5.857	0.004
PALFAMS (range 2–18)	10.69 ± 3.41	8.51 ± 3.64*	7.95 ± 3.72**	8.654	< 0.001

Note: Sex and APOE ε4 status are represented as number (percent), other data are represented as mean ± standard deviation

*NC* Normal control, *SCD* Subjective cognitive decline, *aMCI* amnestic mild cognitive impairment, *MMSE* Chinese Version of the Mini-Mental State Examination, *MoCA-B* Chinese version of the Montreal Cognitive Assessment Basic, *ACE-III* Addenbrooke’s Cognitive Examination III, *AFT* Animal Verbal Fluency Test, *BNT* Boston Naming Test, *STT* Shape Trail Test, *SDMT* Symbol Digit Modalities Test, *AVLT* Auditory Verbal Learning Test, *IR* Immediate Recall, *DR* Delayed Recall, *Re* Recognition, *BVMT-R* Brief Visuospatial Memory Test-Revised, *PLT* Pictorial Learning Test, *DMSPCAD* Delayed Matching to Sample Percent Correct (all delays), *PALFAMS* Paired Associates Learning First Attempt Memory Score

^*^ compared with NC group, *p* < 0.05;** compared with NC group, *p* < 0.01; † compared with SCD group, *p* < 0.05

In the subsequent statistical analysis, all individual neuropsychological test scores were transformed into Z scores to compare the correlations better.

### Correlations of visual memory and other cognitive functions

Based on the results of PCA and clinical experience, we created mean composite scores for the cognitive domains of verbal memory (AVLT-IR, DR, and Recognition [Re]), general cognition (MMSE, MoCA-B, and ACE-III), attention/executive function (STT-A, STT-B, and SDMT), and language function (BNT and AFT). The results of the PCA are shown in Additional file 1.

We then examined the correlations between cognitive domain scores and each visual memory test score. As shown in Table 2, the four visual memory test scores were extensive mildly to moderately correlated with general cognition (r = 0.353–0.502, *p* < 0.01) and verbal memory (r = 0.270–0478, *p* < 0.01) and weakly correlated with language function (r = 0.193–0.262, *p* < 0.05). Although all four tests were claimed to assess visual memory, they are some differences in the components and cognitive domains involved.

**Table 2:** Comparison between visual memory and other cognitive function

	General Cognition	Language Function	Attention/ Executive Function	Verbal Memory
BVMT-R	0.502**	0.262**	-0.107	0.421**
PLT	0.455**	0.232**	-0.138	0.478**
DMS	0.353**	0.247**	-0.069	0.384**
PAL	0.362**	0.193*	-0.133	0.270**

*Note*: *BVMT-R* Brief Visuospatial Memory Test-Revised, *PLT* Pictorial Learning Test, *DMS* Delayed Matching to Sample, *PAL* Paired Associates Learning

^**^p < 0.01,*p < 0.05

### Relative hippocampal subfield volumes differences among groups

MANCOVA showed a statistically significant effect of group on relative hippocampal subfield volumes. In the subgroup analysis by diagnosis, significant effects were found in all relative hippocampal subfield volumes except parasubiculum (F = 5.437–9.815, *p* < 0.005). The subgroup analysis by sex found that men had significant relative hippocampal subregion atrophy (except parasubiculum) than women (F = 4.597–6.699, *p* < 0.034). The subgroup analysis by APOE ε4 status did not yield significance on relative hippocampal subregions (F = 0.008–2.644, *p* > 0.05). There is a significant interaction effect in diagnosis and gene in HATA (F = 3.527, *p* = 0.032). In APOE ε4 positive group, there was a significant decrease in HATA between the aMCI and NC groups (*p* = 0.001) and between the aMCI and SCD groups (*p* = 0.011). In addition, MANCOVA revealed a significance effect of age (except parasubiculum and CA3) (F = 5.978–13.026, *p* < 0.016) but not education level (F = 0.500–3.727, *p* > 0.05) on hippocampal volumes.

Based on MANCOVA results, sex, age, and gene were included as covariates in the ANCOVA. Table 3 shows the statistical results comparing relative hippocampal subfield volumes by diagnosis. ANCOVA controlling for sex, age, and APOE ε4 status did not significantly change the effect of the diagnostic group, except for HATA. The volumes of the CA1, molecular layer, subiculum, GC-ML-DG, CA4, and CA3 were significantly smaller in aMCI patients than in normal controls. However, no significant differences were found between the NC and SCD groups. The volume of the subiculum and presubiculum in the SCD group tended to be larger than in NCs. We also found significant differences in the volume of the CA1, molecular layer, subiculum, presubiculum, GC-ML-DG, and CA4 between the SCD and aMCI groups. The volumes of parasubiculum and HATA did not show differences among the three groups.

**Table 3:** Comparison of relative hippocampal subfield volumes in normal controls and patients with SCD and aMCI

Relative volume	NC	SCD	aMCI	F	p value
CA1	4.28 ± 0.06	4.21 ± 0.07	3.97 ± 0.06**†	7.014	0.001
Molecular-layer	3.78 ± 0.05	3.73 ± 0.06	3.48 ± 0.05**†	9.490	< 0.001
Subiculum	2.92 ± 0.05	2.96 ± 0.05	2.70 ± 0.05**†	8.505	< 0.001
Presubiculum	2.00 ± 0.04	2.05 ± 0.04	1.87 ± 0.04†	5.275	0.006
GC-ML-DG	2.02 ± 0.03	1.95 ± 0.03	1.84 ± 0.03**†	10.374	< 0.001
CA4	1.74 ± 0.02	1.70 ± 0.03	1.61 ± 0.02**†	8.792	< 0.001
CA3	1.40 ± 0.02	1.32 ± 0.02	1.28 ± 0.02**	7.192	0.001
Parasubiculum	0.40 ± 0.01	0.42 ± 0.01	0.41 ± 0.01	0.716	0.491
HATA	0.38 ± 0.01	0.37 ± 0.01	0.36 ± 0.01	2.589	0.079

*Note*: Data are represented as mean ± standard deviation

*NC* normal control, *SCD* subjective cognitive decline, *aMCI* amnestic mild cognitive impairment, *CA* cornu ammonis, *GC-ML-DG* Granule cell molecular layer of dentate gyrus, *HATA* Hippocampal amygdala transition area. Relative hippocampal subfield volumes were calculated by dividing hippocampal subfield volumes by estimated total intracranial volume and multiplying by 10,000. Adjusted for age, sex, and APOE ε4 status

^*^ compared with NC group, *p* < 0.05;** compared with NC group, *p* < 0.01; † compared with SCD group, *p* < 0.05

### Correlation analysis of relative hippocampal subfield volumes and visual memory tasks

In subsequent correlation analyses, only brain structures showing significant differences between the NC, SCD, and aMCI groups (except for the parasubiculum and HATA) were used.

**Fig. 2:**
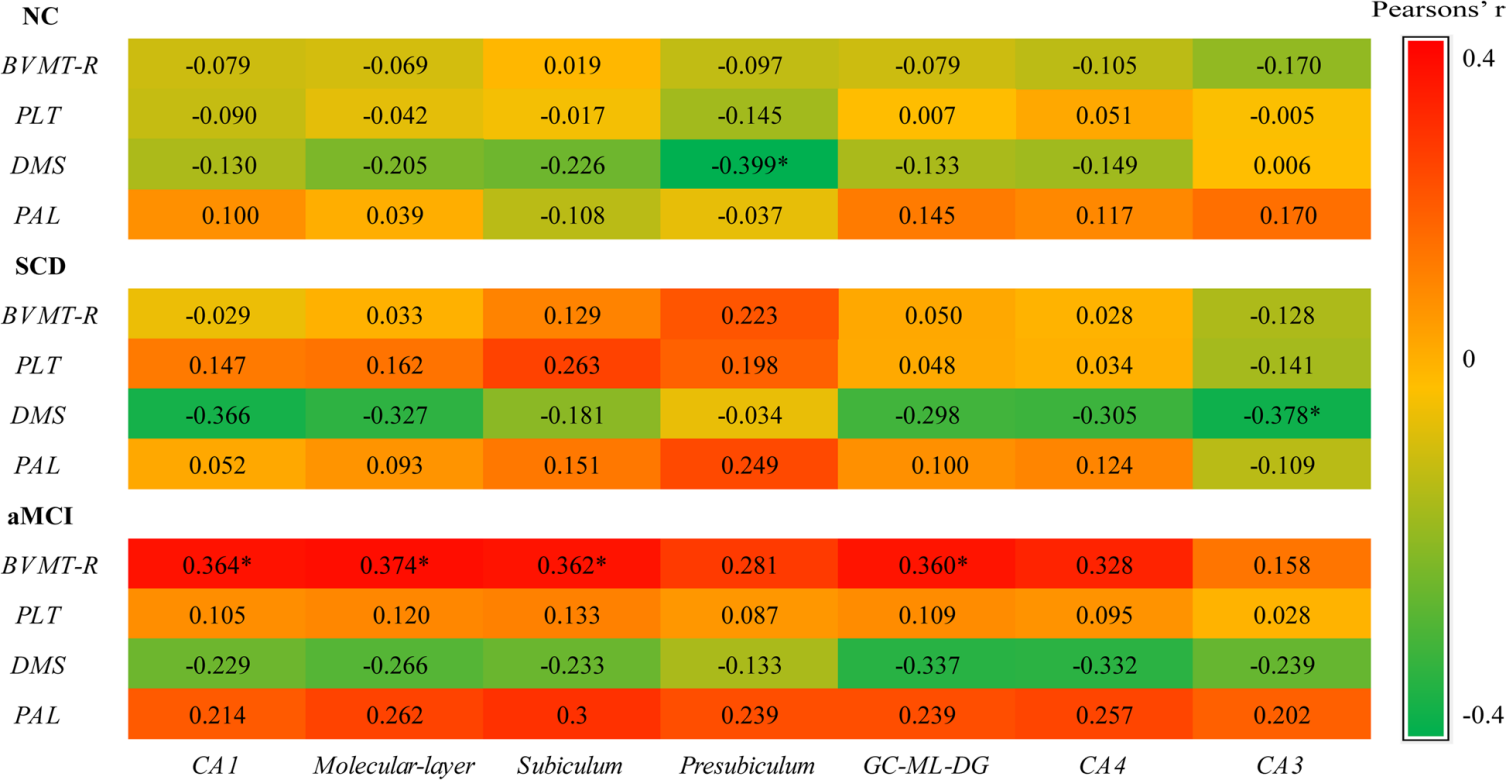
The partial correlation heatmap between relative hippocampal subfield volumes and visual memory scores by diagnosis. NC, normal control; SCD, subjective cognitive decline; aMCI, amnestic mild cognitive impairment; BVMT-R, Brief Visuospatial Memory Test-Revised; PLT, Pictorial Learning Test; DMS, Delayed Matching to Sample; PAL, Paired Associates Learning; CA, cornu ammonis; GC-ML-DG, granule cell molecular layer of dentate gyrus. Adjusted for age, sex, and APOE ε4 status. *FDR (false discovery rate) correction of *p* < 0.05

The correlation heatmap of the partial correlation analysis stratified by diagnosis is included in Fig. 2. Correlation analyses were performed for each group, adjusted for age, sex, and APOE ε4 status, and FDR adjusted for *p* values (q values). In normal controls, we found a significant and negative association between the volume of the presubiculum and DMS delay matching scores (*r* = -0.399, FDR q = 0.024). In the correlation analysis in the SCD individuals, there was a significant and negative correlation between CA3 volume with DMS delay matching scores (*r* = -0.378, FDR q = 0.048). In the aMCI group, moderate and positive correlations were observed between the CA1, molecular layer, subiculum, and GC-ML-DG and BVMT-R immediate recall scores (*r* = 0.360–0.374, FDR q < 0.036).

To ensure robust correlations, we used a stepwise linear regression analysis model to adjust the relationship between MRI brain measurements and cognitive test scores. The majority of the associations mentioned above using Pearson correlation analysis were confirmed in the adjusted models. The DMS delay matching scores in the NC group were negatively associated with the presubiculum volume (β = -0.483, *p* = 0.006). In the SCD group, DMS delay matching scores were negatively associated with the volume of CA3 (β = -0.349, *p* = 0.014). In the aMCI group, the BVMT-R immediate scores were positively associated with the molecular layer (β = 0.416, *p* = 0.006).

## Discussion

The current study examined visual memory and hippocampal subfield volumes in individuals with SCD and aMCI as well as their relationships. We assessed visual memory using four different tests, including immediate memory and delay memory, traditional and computerized tests. SCD showed a slight decline in visual memory performance but no hippocampal subregions atrophy compared with NC. In comparison, aMCI patients had significantly impaired visual memory performance and significant atrophy of the hippocampal subfields. However, the normal controls and patient groups demonstrated different relationships between visual memory performance and hippocampal subfield volumes.

The average onset of hippocampal atrophy was after 60 years [45]. The SCD group in our study showed a decrease in visual memory while the hippocampus remained normal, and we suggested that visual memory may depend not only on the hippocampus but also on the interaction of various complex brain structures, such as the prefrontal cortex and parietal lobes [46]. The previous review reported that approximately half of clinical-based studies found normal hippocampal volumes in SCD patients [15] and suggested that SCD was associated with longitudinal hippocampal atrophy [17]. When we looked closely at the SCD results, there was a trend towards increased volumes in the presubiculum and subiculum. One study found atrophy of these two regions was suggested to be the earliest hippocampal anatomical markers of Alzheimer’s disease [44]. A previous longitudinal study found that larger hippocampal volumes are related to greater increases in recall performance after cognitive intervention for subjective memory impairment [47]. Other researchers considered enlargement as a compensatory mechanism that protects memory decline and enhances memory persistence [48].

As expected, significant memory impairment and extensive atrophy of the hippocampal subregions were observed in the aMCI group, which was consistent with the previous study [16, 49]. There are still controversies about which hippocampal subfield is the most reliable for distinguishing NC from aMCI/AD and predicting conversion. Several studies have suggested that CA1 atrophy may be a biomarker for preclinical AD because of the earliest effects of neurofibrillary tangles and neuronal loss [18, 50]. Other studies found the subiculum and presubiculum accurately classify AD from NC and predict the conversion of MCI to AD [44, 51, 52]. Some researchers also found that DG-CA3 was more sensitive to associative memories than to other divisions of the hippocampus [53] and were crucial factors influencing the formation and storage of situational memories [54].

We found that visual memory was negatively associated with presubiculum in NC. While in the SCD group, a smaller CA3 volume was associated with better delay memory performance. There were broad related hippocampal subfields (CA1, molecular-layer, subiculum, GC-ML-DG) in aMCI. The different relationships between visual memory performance and hippocampal subfield volumes are reflected in different hippocampal subfields and visual memory tests. The correlations involved more subfields as cognitive decline. The researcher found that the correlation patterns between anatomical volumes and memory scores depended greatly on the pattern of hippocampal atrophy [44]. However, the correlation patterns in healthy participants and SCD patients have been complicated to interpret and have produced a somewhat equivocal pattern of findings [55]. The relevant brain regions of NC and SCD were probably considered because of their better predictive value. Previous studies found positive (subiculum and CA1 with delayed visual recall) [27], negative (left hippocampal with free word recall) [19], or no [56] correlations between hippocampus/hippocampal subfields and memory in healthy participants. A positive correlation was found between right hippocampal volumes with free and delayed recall memory in an SCD group [19]. Significant positive correlations were found between volumes of CA2/3, CA4–DG, and the subiculum complex and immediate and delayed recall in aMCI and AD [16, 27, 44]. We suggest that these bidirectional correlations are associated with nonlinear and heterogeneous changes in different stages of hippocampal subregions. A compensatory volume increase may explain a negative correlation to maintaining memory. Functional imaging studies suggested that hippocampal subfields may have different involvement in memory over different time scales [57]. Whether these predict trends in hippocampal subregions at different stages needs longitudinal methods.

Of course, this discrepancy pattern could also be due to various ways of measuring cognition or subject differences. DMS mainly assesses short-term visual memory, while BVMT-R measures visual memory and spatial relationships. In the present study, DMS lacked the sensitivity to measure visual memory in the SCD group and had weaker associations with each cognitive domain than BVMT-R. Nevertheless, it was the only variable associated with the hippocampus in SCD and NC. We suppose DMS may measure other cognitive domains not included in the traditional neuropsychological battery [58]. We found hippocampus-related memory test was BVMT-R immediate recall but not PLT delay recall in aMCI. Previously, BVMT-R immediate recall/learning had a stronger correlation with the hippocampus than BVMT-R delay recall in aMCI [28]. Nevertheless, hippocampus and hippocampal subregions are often more strongly linked with delayed than immediate memory [26, 59]. We consider meaningless graphics in BVMT-R and DMS to involve more learning than a storage function [56]. Future need perform molecular biology experiments to explore the relationship between this brain region and memory.

This study design has several limitations. First, the cross-sectional study could not determine a cause-and-effect relationship between neuroimaging changes and an individual's cognitive ability. Follow-up evaluations in these participants are needed. In addition, we did not include Aβ markers in this study, so the actual extent of AD pathology remains unknown. Third, it is essential to note that although internally validated from high-field data, the FreeSurfer segmentations do not necessarily represent ground truth in anatomy and must be interpreted with caution [60]. Last, the average left and right hippocampus may ignore laterality.

## Conclusions

In conclusion, the evidence from this study indicates that extensive atrophy of hippocampal subfields in aMCI relative to SCD and healthy elders. No significant hippocampal subfields differences were found between controls and SCD. After adjustment, our data support an intrinsic connection between structural imaging and visual memory in three groups. These results suggest that the preclinical stage of AD is a highly variable and complex process. The different correlation patterns may indicate that the procession of brain changes during NC to SCD then aMCI. Future work should combine both structural and functional data to investigate the associations between hippocampal subfield patterns, corresponding functional signatures, and cognitive performance, which would help tease out how these variables interact over time.

## Supplementary Information

**Additional file 1.** Principal components analysis of neuropsychological tests.

## Data Availability

The datasets generated and/or analysed during the current study are not publicly available due to privacy and data sharing issues but are available from the corresponding author on reasonable request.

## Abbreviations

AD: Alzheimer’s disease
aMCI: Amnestic mild cognitive impairment
Aβ: Amyloid beta
SCD: Subjective cognitive decline
PAL: Paired Associates Learning
DMS: Delayed Matching To Sample
BVMT-R: Brief Visuospatial Memory Test-Revised
CA: Cornu ammonis
DG: Dentate gyrus
AVLT: Auditory Verbal Learning Test
HVLT-R: Hopkins Verbal Learning Test-Revised
NC: Normal control
MMSE: Chinese Version Of The Mini-Mental State Examination
MoCA-B: Chinese version of the Montreal Cognitive Assessment Basic
CDR: Clinical Dementia Rating
SD: Standard deviation
PLT: Pictorial Learning Test
CANTAB: Cambridge Neuropsychological Test Automated Battery
IR: Immediate Recall
DR: Delayed Recall
DMSPCAD: Delayed Matching To Sample Percent Correct (All Delays)
PALFAMS: Paired Associates Learning First Attempt Memory Score
ACE-III: Addenbrooke’s Cognitive Examination III
AFT: Animal Verbal Fluency Test
BNT: Boston Naming Test
STT: Shape Trail Test
SDMT: Symbol Digit Modalities Test
GC-ML-DG: Granule cell molecular layer of dentate gyrus
HATA: Hippocampal amygdala transition area
eTIV: Estimated total intracranial volume
PCA: Principal component analysis
MANCOVA: Multivariate analysis of covariance
ANCOVA: Analysis of covariance
FDR: False discovery rate
Re: Recognition
