# Supplementary material for: Differential associations of visual memory with hippocampal subfields in subjective cognitive decline and amnestic mild cognitive impairment
Source: BMC Geriatr. 2022 Feb 24;22:153. doi: 10.1186/s12877-022-02853-7 (PMC8876393; doi:10.1186/s12877-022-02853-7)
Supplement: Supplementary file 1 — Additional file 1. Principal components analysis of neuropsychological tests. [file 12877_2022_2853_MOESM1_ESM.docx]

Additional file 1

Principal components analysis of neuropsychological tests

| Subtest | Components | | |
| --- | --- | --- | --- |
|  | 1 | 2 | 3 |
| % of variance | 46.278 | 11.407 | 9.003 |
| ACE-Ⅲ | **0.827** | 0.241 | 0.224 |
| MMSE | **0.82** | 0.045 | 0.037 |
| AVLT-IR | **0.771** | 0.039 | 0.195 |
| AVLT-DR | **0.75** | 0.189 | 0.144 |
| AVLT-Re | **0.727** | 0.283 | 0.209 |
| MoCA-B | **0.666** | 0.321 | 0.209 |
| STT-A | -0.026 | **-0.836** | -0.136 |
| STT-B | -0.234 | **-0.787** | -0.075 |
| SDMT | 0.459 | **0.546** | 0.083 |
| BNT | 0.097 | 0.128 | **0.887** |
| AFT | 0.393 | 0.115 | **0.69** |

Note: ACE-III, Addenbrooke’s Cognitive Examination III; MMSE, Chinese Version of the Mini-Mental State Examination; AVLT, Auditory Verbal Learning Test; IR, Immediate Recall; DR, Delayed Recall; Re, Recognition; MoCA-B, Chinese version of the Montreal Cognitive Assessment Basic; STT, Shape Trail Test; SDMT, Symbol Digit Modalities Test; BNT, Boston Naming Test; AFT, Animal Verbal Fluency Test.
